# Treatment of cartilage defects in the patellofemoral joint with matrix-associated autologous chondrocyte implantation effectively improves pain, function, and radiological outcomes after 5–7 years

**DOI:** 10.1007/s00402-023-05179-0

**Published:** 2024-01-11

**Authors:** Martin Eichinger, Benjamin Henninger, Benjamin Petry, Philipp Schuster, Elmar Herbst, Moritz Wagner, Ralf Rosenberger, Raul Mayr

**Affiliations:** 1 Department of Orthopaedics and Traumatology, a.ö. Bezirkskrankenhaus St. Johann in Tirol, Bahnhofstraße 14, 6380 St. Johann in Tirol, Austria; 2 Department of Orthopaedics and Traumatology, Medical University of Innsbruck, Innsbruck, Austria; 3 Department of Radiology, Medical University of Innsbruck, Innsbruck, Austria; 4 Department of Orthopaedic Surgery, Waikato Hospital, Hamilton, New Zealand; 5 Department of Sports Orthopaedics and Special Joint Surgery, RKH Orthopaedic Hospital, Markgröningen, Germany; 6 Department of Orthopaedics and Traumatology, Paracelsus Medical University, Clinic Nuremberg, Nuremberg, Germany; 7 Department of Trauma, Hand and Reconstructive Surgery, University of Münster, Münster, Germany; 8 Privatklinik Hochrum, Sanatorium Der Kreuzschwestern, Rum, Austria

**Keywords:** Cartilage, Chondral defect, Patellofemoral joint, Matrix-associated autologous chondrocyte implantation, Clinical outcomes, Magnetic resonance observation of cartilage repair tissue score

## Abstract

**Introduction:**

The aim of the present study was to evaluate midterm outcomes 5–7 years after matrix-associated autologous chondrocyte implantation (MACI) in the patellofemoral joint.

**Materials and methods:**

Twenty-six patients who had undergone MACI using the Novocart® 3D scaffold were prospectively evaluated. Clinical outcomes were determined by measuring the 36-Item Short-Form Health Survey (SF-36) and International Knee Documentation Committee (IKDC) scores and the Western Ontario and McMaster Universities Osteoarthritis Index (WOMAC) values preoperatively and 3, 6, and 12 months, and a mean of 6 years postoperatively. At the final follow-up, the Magnetic Resonance Observation of Cartilage Repair Tissue (MOCART) score was evaluated.

**Results:**

Twenty-two patients with 23 focal cartilage defects (19 patella and four trochlea) were available for the final follow-up. The mean defect size was 4.0 ± 1.9 cm^2^ (range 2.4–9.4 cm^2^). All clinical outcome scores improved significantly until 5–7 years after MACI (SF-36 score, 61.2 ± 19.6 to 83.2 ± 11.6; *P* = 0.001; IKDC score, 47.5 ± 20.6 to 74.7 ± 15.5; *P* < 0.001; and WOMAC, 29.8 ± 15.7 to 8.2 ± 10.3; *P* < 0.001). The mean MOCART score was 76.0 ± 11.0 at the final follow-up. Nineteen of the 22 patients (86.4%) were satisfied with the outcomes after 5–7 years and responded that they would undergo the procedure again.

**Conclusion:**

MACI in the patellofemoral joint demonstrated good midterm clinical results with a significant reduction in pain, improvement in function, and high patient satisfaction. These clinical findings are supported by radiological evidence from MOCART scores.

**Level of evidence:**

IV–case series.

## Introduction

Cartilage defects in the knee joint cause significant pain and disability, leading to osteoarthritis in many cases [1, 2]. Defects in the patellofemoral (PF) joint account for one-third of knee cartilage defects and pose a unique challenge for orthopedic surgeons. This is due to the complex biomechanics of the PF joint, which involve the occurrence of shear forces that can be particularly detrimental to cartilage tissue [3]. Therefore, the treatment of PF cartilage defects requires careful consideration and an appropriate approach. Matrix-associated autologous chondrocyte implantation (MACI) has become the preferred treatment method for large cartilage defects (> 2 cm^2^), with the potential for significant long-term socioeconomic benefits [4, 5]. MACI aims to create hyaline-like cartilage tissue to reduce pain and restore function in everyday life, work, and sports in affected patients over the long term. However, evaluation of clinical outcomes is essential for assessing the efficacy and success of MACI, and noninvasive magnetic resonance imaging (MRI) assessments have provided valuable insights into the quality and morphology of the cartilage repair tissue [6–11].

For MACI in the PF joint, however, comprehensive prospective mid- and long-term data, especially clinical data with detailed MRI scores, are currently lacking [9–12]. Thus, the aim of the present study was to prospectively evaluate clinical and MRI outcomes over a minimum of 5 years following MACI using the Novocart® 3D matrix for PF cartilage defects. Outcomes were assessed using three clinical and one MRI-based validated and well-established scores [10, 13–15]. The hypotheses were that MACI would yield significant improvements in clinical outcome measures (1) and show good repair tissue quality in radiological assessments (2) in a midterm follow-up.

## Materials and methods

Patients who were treated with MACI using the Novocart® 3D scaffold at the authors’ institution between 2008 and 2011 were prospectively followed up if they met the inclusion criteria, i.e., age between 18 and 49 years with symptomatic International Cartilage Repair Society (ICRS) grade III and IV cartilage defects > 2 cm^2^ of the patellofemoral aspect of the knee joint with a regular mechanical axis (< 5° malalignment). The exclusion criteria were unaddressed instabilities of the knee joint, previous (sub)total meniscectomy, arthrofibrosis, metabolic arthritis, rheumatologic, autoimmune, inflammatory, and other comorbidities influencing cartilage metabolism, severe neurological disorders or psychological diseases, pregnancy, as well as contraindications against MRI.

The study was approved by the local ethics board, and all patients provided written informed consent. The final follow-up examination was performed after an average of 6 years.

### Surgical procedure

#### First intervention—arthroscopic cartilage harvesting

In the first intervention, arthroscopic harvesting of hyaline cartilage autografts from the non-weight bearing aspect of the intercondylar notch of the ipsilateral knee was performed. Depending defect size, 2 or 4 cylinders were harvested, targeting a cell density of 0.75 to 4 million cells/cm^2^ for the Novocart® 3D and Novocart® 3D XL matrices.

These autografts were cultured for three weeks, during which chondrocytes were isolated, multiplied, and seeded onto the biphasic, collagen type I-based Novocart® 3D scaffold to contain 8.25–44 million cells with over 95% vitality.

#### Second intervention—implantation

**Fig. 1:**
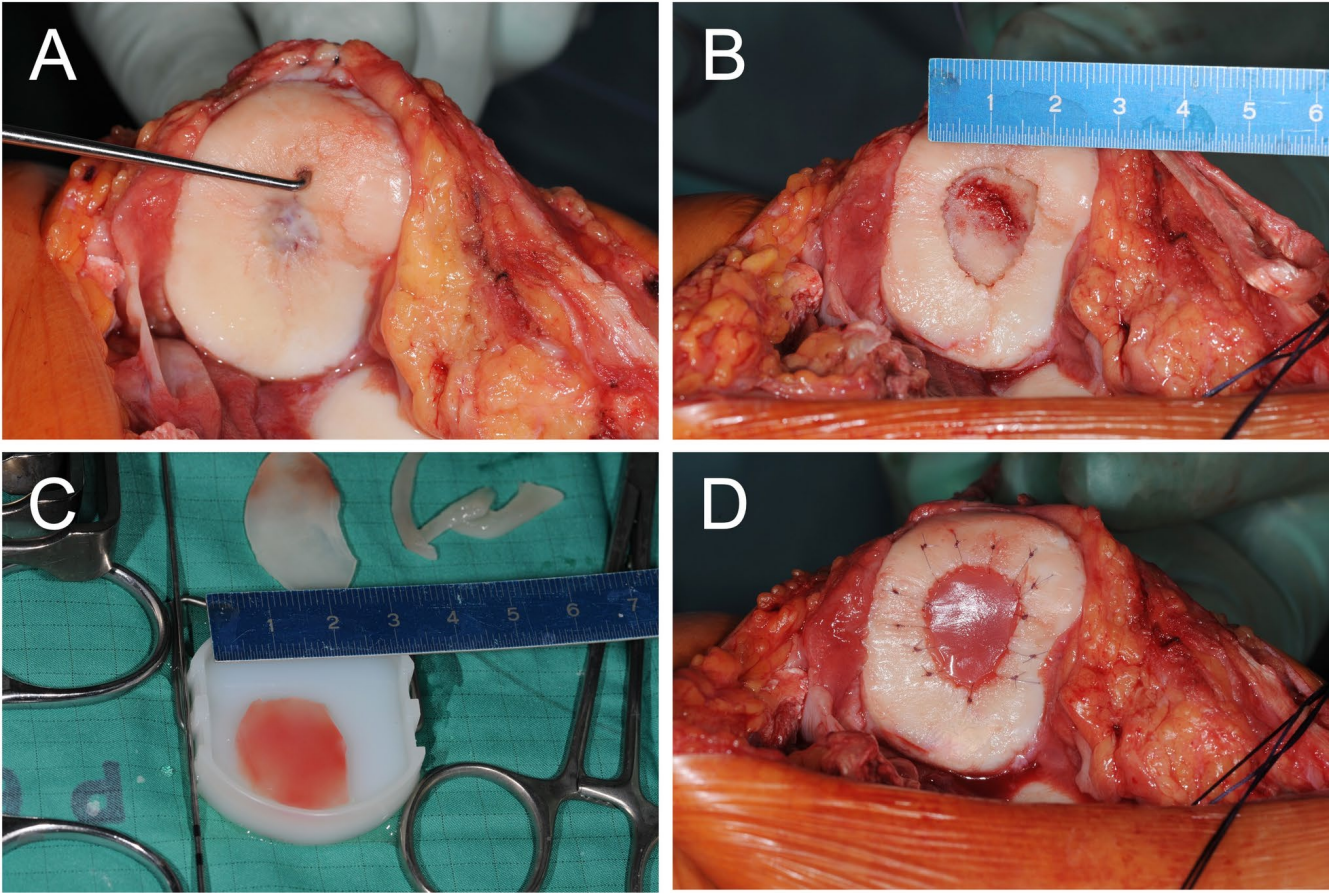
Intraoperative images of matrix-associated autologous chondrocyte implantation using the Novocart® 3D scaffold in a retropatellar defect with concomitant medial patellofemoral ligament reconstruction in a 43-year-old male patient. **A** Probing of the affected cartilage surrounding the defect. **B** Full size of the prepared defect after resection of all affected cartilage tissue until a stable ream was obtained. **C** Matrix cut to size. **D** Transplantation in situ

The cartilage defects were visualized by mini/arthrotomy. After debridement of the defect was performed the matrix was inserted in the defect with the porous side facing the lamina and fixed with resorbable sutures and fibrin glue. (Fig. 1).

### Rehabilitation

Rehabilitation was conducted in accordance with a standardized protocol for autologous chondrocyte implantation of the PF joint [16]. During the acute phase, special attention was paid to the consequent cryotherapy of the knee. Bed rest and immobilization in full extension during the first postoperative day were followed by early continuous passive mobilization, which was continued daily for the first eight weeks (ideally 6–8 h per day). Patients were encouraged to weight-bear to tolerance in extension. The ideal range of motion (ROM) was guaranteed using an orthopedic brace set to 0–0–30° for the first two postoperative weeks, 0–0–60° for weeks 3–4, and 0–0–90° for weeks 5–6, after which the brace was removed. In the initial rehabilitation phase (weeks 1–6), patients engaged in self-assisted range of motion (ROM) exercises, cross-education, and proprioceptive neuromuscular facilitation. Subsequent weeks focused on restoring full ROM, normal gait, and strength through closed kinetic chain exercises. From 3 to 6 months post-operation, moderate sports like cycling, swimming, and Nordic walking were permitted. Impact sports were not allowed before 12 months postoperatively.

### Clinical evaluation

Clinical outcome was evaluated by three validated outcome scores for the knee joint, i.e., the 36-Item Short-Form Health Survey (SF-36) [15] score, the International Knee Documentation Committee (IKDC) score [14], and the Western Ontario and McMaster Universities Osteoarthritis Index (WOMAC) Likert version 3.1 with the subscales for pain, joint stiffness, and physical function in daily activities [13]. These scores were transformed to a 0–100 scale. Thus, all results of the clinical evaluation ranged from 0 to 100, with higher SF-36 and IKDC scores indicating better outcomes and lower WOMAC index values and subscale scores indicating better overall outcomes with less pain, less stiffness, and less impairment of physical function in daily activities. The clinical scores were collected preoperatively, after 3, 6, and 12 months, and 5–7 years postoperatively.

### Radiological evaluation

**Fig. 2:**
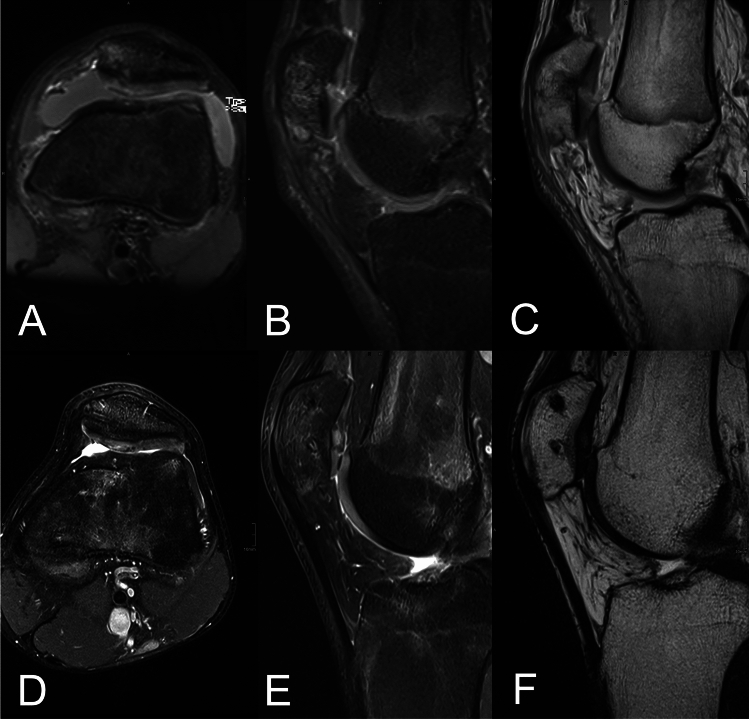
Typical magnetic resonance imaging scans of a 19-year-old male patient with a retropatellar cartilage defect ICRS grade IV with subchondral reaction and joint effusion: **A** axial PD-weighted with fat saturation, **B** sagittal PD-weighted with fat saturation, and **C** sagittal PD-weighted sequences. MRI scans obtained 5 years after MACI with Novocart® 3D and concomitant medial patellofemoral ligament reconstruction showing complete filling of the defect with complete integration, intact surface of the repair tissue, and an inhomogeneous structure. The subchondral lamina and bone are intact, with no adhesions or effusion: **D** axial PD-weighted with fat saturation, **E** sagittal PD-weighted with fat saturation, **F** and sagittal T2-weighted sequences

Special cartilage-sensitive high-resolution MRI for measurement of radiological outcomes was performed at the final follow-up (Fig. 2).

#### MRI specifications

All MRI examinations were performed with a 1.5-T whole-body MR scanner (MAGNETOM Avanto^fit^; Siemens Healthineers). Patients were scanned in the supine position, feet first, using a dedicated 15-channel knee coil. The MRI protocol consisted of the following sequences: (1) coronal T1-weighted (echo time [TE], 9.4 ms; repetition time [TR], 500–704 ms; slice thickness [SL] 3 mm), (2) sagittal PD-weighted (TE, 39 ms; TR, 3620–4600 ms; SL, 3 mm), (3) coronal PD-weighted (TE, 41 ms; TR, 3990–4580 ms; SL, 3 mm), (4) axial PD-weighted (TE, 37 ms; TR, 3500–4280 ms; SL, 3.5 mm), (5) sagittal PD-/T2-weighted dual-echo (TE, 33/89 ms; TR, 3840-4410 ms; SL, 3 mm).

#### Magnetic resonance observation of cartilage repair tissue

A trained radiologist with over 10 years of experience in musculoskeletal radiology analyzed the images using the imaging viewer Impax EE (Agfa Health Care N.V., Mortsel, Belgium). The Magnetic Resonance Observation of Cartilage Repair Tissue (MOCART) score was assessed for all patients [10].

### Statistical analysis

Normal distribution of the data was confirmed with the Shapiro–Wilk test. One-way repeated measures of variance (ANOVA) was used to evaluate changes in clinical outcome measures over time. Paired *t* tests were calculated for comparison of pre-and postoperative outcome scores. Pearson’s *ρ* was used for evaluating the correlation of MOCART and clinical outcome scores. Statistical significance was defined as a *P* value < 0.05 with a two-sided 95% confidence interval. Statistical analysis was performed with IBM SPSS Statistics for Mac version 27.0 (IBM Corp, Armonk, New York, USA).

## Results

Of the 26 patients included in this study, four were lost to follow-up (follow-up rate, 85%). Thus, 23 PF cartilage defects of 22 patients (5 female and 17 male) aged between 18 and 49 years (mean age, 29.5 ± 11.0 years) were evaluated at the final follow-up examination after 5–7 years. Nineteen defects were located at the retropatellar surface and four were located in the trochlea; 22 defects were categorized as ICRS grade 4 and one was categorized as a grade 3 lesion. The cartilage lesions resulted from injuries during sports, everyday life, or recurrent patellar instability. Nine cases involved previous surgeries, including removal of loose bodies, debridement, plica resection, partial meniscectomy, patella-stabilizing procedures, anterior cruciate ligament reconstruction, and osteosynthesis of the patella. None of the defects had been treated with prior microfracturing. Concomitant surgeries were performed in nine patients, including one partial meniscectomy and nine corrective interventions for patellar alignment (Table 1). The mean relative cell count of the implanted matrices was 1.83 ± 0.71 million cells per cm^2^ (range 0.79–3.64 million cells per cm^2^).

**Table 1:** Descriptive data of the patient cohort

Characteristics	Values
Age, (years)	29.5 ± 11.0 (18–49)
Sex female/male, (*n*)	5/17
Knee right/left, (*n*)	12/10
Defect localization, (*n*)	
Patella	19
Trochlea	4
Defect size, (cm^2^)	4.0 ± 1.9 (2.4–9.4)
ICRS defect grade III/IV, (*n*)	1/22
Concomitant surgeries (*n*)	
MPFL reconstruction	5
Transfer of tibial tuberosity	2
VMO transfer	1
Partial meniscectomy	1

Data are shown as mean with standard deviation, (range) and absolute numbers (*n*)

*ICRS* International Cartilage Repair Society, *MPFL* medial patellofemoral ligament, *VMO* vastus medialis obliquus

Typical postoperative swelling and effusion resolved in all patients within the first months after surgery. None of the patients showed infection or detachment of the transplants. Four revision surgeries were performed: one scar revision due to troublesome parapatellar adhesions that were resolved by local scar release 8 months after implantation, and three transplant hypertrophies that were treated with arthroscopic trimming 4, 10, and 21 months after implantation.

### Clinical outcomes

The health-related quality of life, as measured by the SF-36 score, showed a significant improvement over time (*P* < 0.001) with a high effect size (*η*_*p*_^2^ = 0.65). The SF-36 score increased from 61.2 ± 19.6 preoperatively to 83.2 ± 11.6 at 5–7 years postoperatively (*P* < 0.001).

The patients' preoperative symptoms included pain, effusion, and reduced knee function, which were reflected in their preoperative mean IKDC score of 47.5 ± 20.8 and WOMAC index of 29.8 ± 15.7. The clinical outcome measures, i.e., the IKDC score and WOMAC index, improved significantly from baseline to the final 5–7-year follow-up, reaching 74.7 ± 15.5 and 8.2 ± 10.3, respectively (*P* < 0.001). The improvements in both the IKDC score and WOMAC index throughout the follow-up period were statistically significant (*P* < 0.001) with a high effect size (*η*_*p*_^2^ = 0.68 and *η*_*p*_^2^ = 0.75, respectively).

**Fig. 3:**
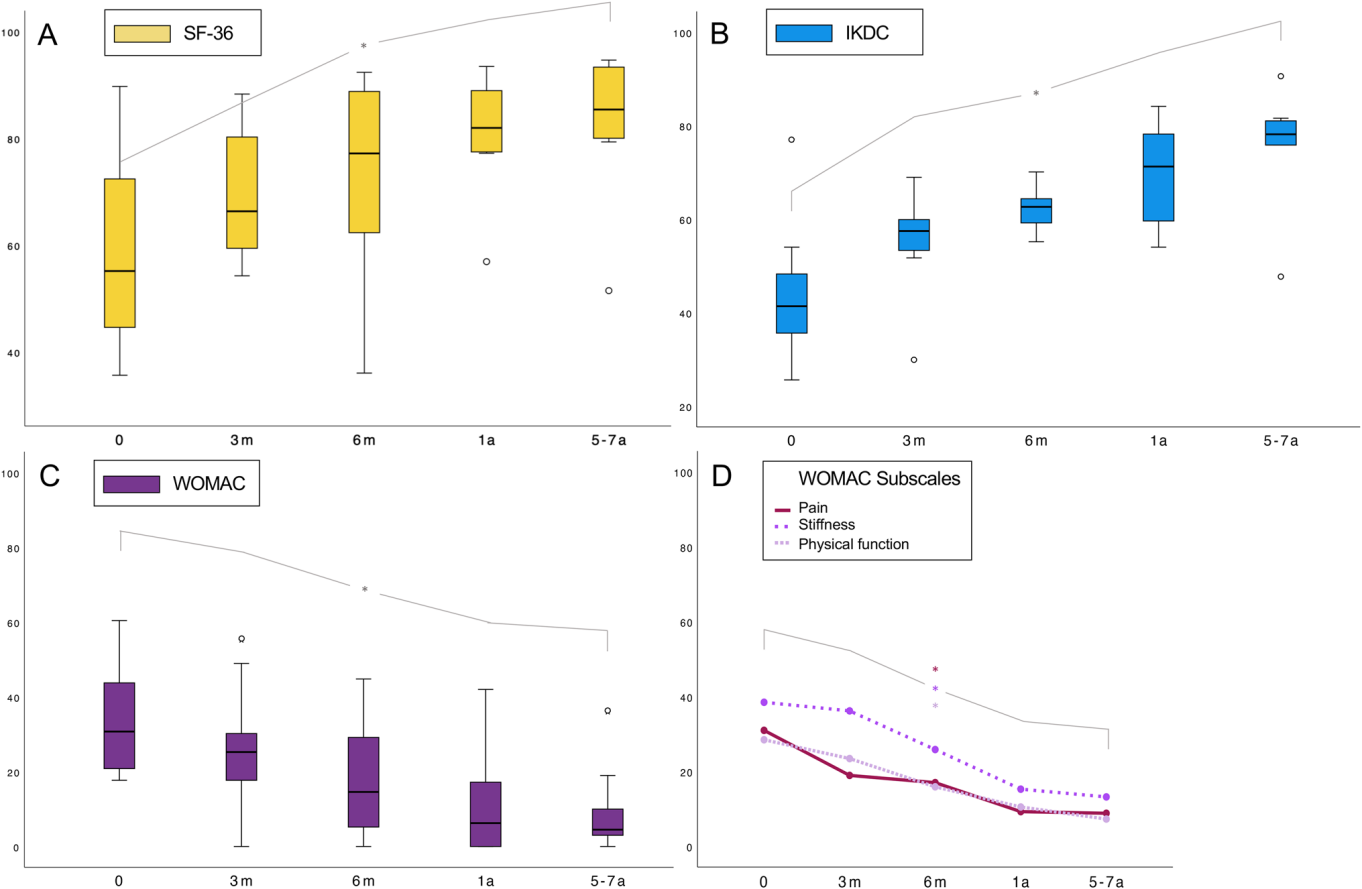
Boxplot of clinical outcome scores throughout the follow-up period. **A** Boxplot of Short-Form Health Survey 36 (SF-36) scores. **B** Boxplot of International Knee Documentation Committee (IKDC) scores. **C** Boxplot of the Western Ontario and McMaster Universities Osteoarthritis (WOMAC) Index. **D** Line diagram of WOMAC subscale scores for pain, stiffness, and physical function in daily activities. All scores were transformed to a 0–100 scale; for IKDC and SF-36, higher scores indicate better outcomes; for the WOMAC index and subscale scores, lower values indicate better overall outcomes with less pain, stiffness, and impairment of physical function in daily activities. *Indicates statistically significant differences throughout the follow-up period

The pain decreased to a low level throughout the follow-up period, as documented by the WOMAC subscale score for pain, which improved significantly from 29.8 ± 15.7 preoperatively to 9.0 ± 10.1 after 5–7 years (*P* < 0.001). The subscale scores for joint stiffness and function in daily activities also improved significantly from 38.5 ± 25.7 and 28.5 ± 16.3 to 13.9 ± 16.0 and 7.4 ± 10.3, respectively (*P* = 0.003 and *P* < 0.001, respectively). Table 2 and Fig. 3 provide a detailed overview of the absolute scores for each clinical outcome measure at the respective follow-up timepoints.

**Table 2:** Clinical results: SF-36 and IKDC scores and WOMAC index and subscale scores

Follow-up (months)	SF-36 score	IKDC score	WOMAC	WOMAC subscale values		
				*P*	JS	DFA
0	61.2 ± 19.6	47.5 ± 20.6	29.8 ± 15.7	31.0 ± 15.3	38.5 ± 25.7	28.5 ± 16.3
3	70.3 ± 13.2	54.2 ± 17.2	23.5 ± 16.8	18.9 ± 17.2	36.1 ± 24.3	23.4 ± 17.1
6	73.3 ± 16.1	57.8 ± 11.9	16.9 ± 13.2	17.0 ± 14.5	25.8 ± 17.3	15.9 ± 13.8
12	80.1 ± 10.2	70.6 ± 10.7	10.5 ± 12.7	9.3 ± 10.7	15.3 ± 15.1	10.5 ± 12.6
60–84	83.2 ± 11.6	74.7 ± 15.5	8.2 ± 10.3	9.0 ± 10.1	13.9 ± 16.0	7.4 ± 10.3
*P *(ANOVA time)	< 0.001	< 0.001	< 0.001	0.006	< 0.001	< 0.001
*η*_*p*_^2^ (effect size)	0.65	0.68	0.75	0.65	0.77	0.49
*P *(*t* test 0 vs. 60–84 months)	0.001	< 0.001	< 0.001	< 0.001	0.003	< 0.001

Data are shown as means with standard deviation

*SF-36* Short-Form Health Survey 36, *IKDC* International Knee Documentation Committee, *WOMAC* Western Ontario and McMaster Universities Osteoarthritis Index. WOMAC Subscales: *P* pain, *JS* joint stiffness, *DFA* daily functional activities, *ANOVA* analysis of variance

### Radiological outcome

At the final follow-up, the mean MOCART score was 76.0 ± 11.0 (range 47–88; Fig. 2).

None of the patients had an effusion. MRI scans showed signal alteration in the subchondral bone in 16 patients (69.6%), while the subchondral lamina was intact in 12 patients (52.2%). The structural signal of the repair tissue was altered in 16 patients (69.6%), but the surface of the repair tissue was rated as intact in 14 patients (60.9%). Most patients showed complete integration of the transplant to the border zone (11 cases, 47.8%) or a visible demarcating border (nine cases, 39.1%), while incomplete integration of less than 50% of the length was observed in only three cases (13.0%). MRI scans in most patients (18 cases, 78.3%) showed a complete fill of the defects at the final follow-up. Only three patients (13.0%) showed an incomplete fill with greater than 50% fill of the defect, and two patients (8.7%) showed asymptomatic hypertrophy of the transplant. For more detailed information on the MRI evaluation and MOCART parameters, please refer to Table 3.

**Table 3:** Radiological results: MOCART scores after 5–7 years

Parameters	Points	*N*	(%)
		of defects	
Degree of defect repair and filling of the defect			
Complete fill	20	18	(78.3)
Hypertrophy	15	2	(8.7)
Incomplete fill > 50%	10	3	(13.0)
Incomplete fill < 50%	5	0	(0)
Cartilage interface/integration to the border zone			
Complete	15	11	(47.8)
Demarcating border visible	10	9	(39.1)
Defect < 50% (of length)	5	3	(13.0)
Defect > 50% (of length)	0	0	(0)
Surface of the repair tissue			
Intact	10	14	(60.9)
Damaged < 50% of depth	5	9	(39.1)
Damaged > 50% of depth	0	0	(0)
Structure of the repair tissue			
Homogeneous	5	7	(30.4)
Inhomogeneous or cleft formation	0	16	(69.6)
Signal intensity on dual T2-FSE			
Isointense	15	13	(56.5)
Moderately hypo-/hyperintense	10	10	(43.5)
Markedly hypo-/hyperintense	0	0	(0)
Subchondral lamina			
Intact	5	12	(52.2)
Not intact	0	11	(47.8)
Subchondral bone			
Intact	5	7	(30.4)
Not intact	0	16	(69.6)
Adhesions			
No	5	15	(65.2)
Yes	0	8	(34.8)
Effusion			
No	5	23	(100)
Yes	0	0	(0)

*MOCART* Magnetic Resonance Observation of Cartilage Repair Tissue, *FSE* fast spin echo

**Fig. 4:**
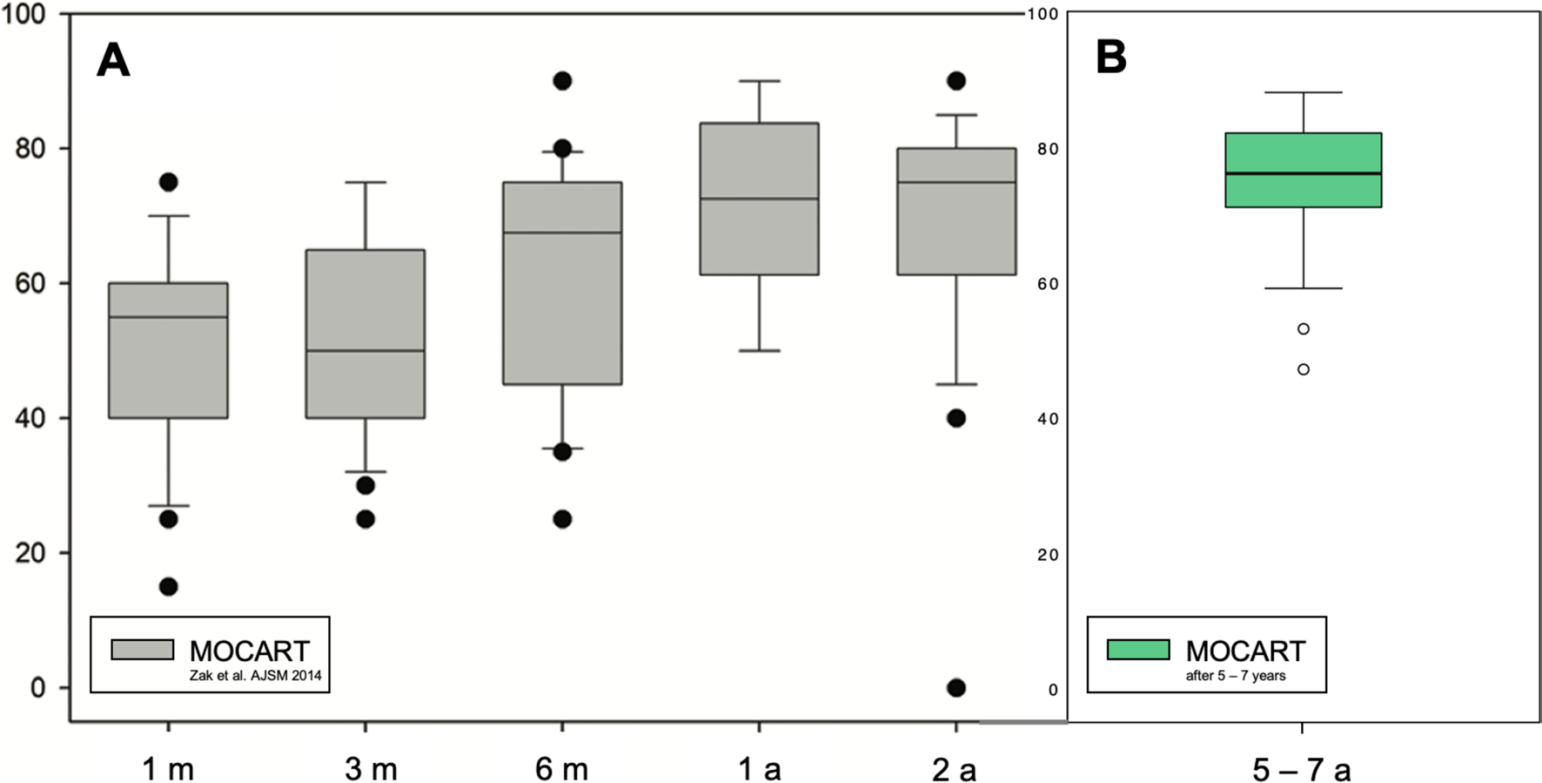
Boxplot of **A** magnetic resonance observation of cartilage tissue (MOCART) scores in a comparable cohort of 23 patients (25 Novocart® 3D transplants) with a close radiological follow-up between 1 and 24 months published by Zak et al. 2014 AJSM [33] – reprinted with permission of the authors and the publisher, copyright: SAGE; **B** MOCART scores in our cohort of 22 patients (23 Novocart® 3D transplants) 5–7 years after surgery

No statistically significant correlation was observed between MOCART and clinical outcome scores at the 5–7-year follow-up (*P*-values ranging from 0.158 to 0.869) (Fig. 4).

## Discussion

The most important finding of this study was the good clinical outcomes of MACI in the PF joint, which showed significant improvements during a follow-up period of 5–7 years, confirming the first hypothesis. The MRI analysis supported the second hypothesis, demonstrating a high rate of complete defect filling and high MOCART scores.

Previous studies on PF cartilage repair using third-generation MACI are rare, particularly with follow-up periods of five years or longer [3, 17, 18]. Most studies with long-term follow-up data focused on first-generation autologous chondrocyte implantation (ACI) or MACI with mixed cohorts involving all knee compartments and various scaffolds [6, 7, 19–23]. The present study specifically examined MACI in the PF joint and observed significant and sustained improvements in all clinical outcome measures. These improvements were maximal by one year and persisted throughout the follow-up period, with all of the scores surpassing the minimal clinically important difference threshold. These are encouraging findings for the use of MACI to treat PF cartilage defects.

Addressing maltracking and instability of the PF joint is often necessary in conjunction with MACI, and the results of the present study align with the growing evidence suggesting that these factors do not negatively influence the outcomes of PF MACI if they are adequately addressed [18, 24–26]. Consistent with the literature, various concomitant procedures, including medial patellofemoral ligament reconstruction, vastus medialis obliquus transfer, lateral retinaculum lengthening, and transfer of tibial tuberosity, were performed in the present cohort.

Another potential concern with MACI in the PF joint is an increased risk of graft hypertrophy (GH), particularly in patellar cartilage repair [27–29]. In the current study a similar percentage of GH (symptomatic, 13.6%; asymptomatic, 9.1%) was observed as described in the literature [27–31]. Although three patients required arthroscopic graft trimming, evidence suggests that GH in third-generation MACI may often remain asymptomatic or diminish over time [28, 29].

Notwithstanding the challenging factors associated with treating PF cartilage defects, the present study demonstrates promising clinical outcomes with MACI in the PF joint. Significant improvements were observed in the SF-36 and IKDC scores and the WOMAC index when comparing the preoperative values with those obtained 5–7 years postoperatively. The most substantial gains were noted between the 6- and 12-month assessments. Overall patient-satisfaction level was high, with 86.4% of patients stating they were both satisfied with the outcome after 5–7 years and would undergo the procedure again if necessary.

Previous studies have reported similarly favorable clinical outcomes after MACI in the PF joint. Niethammer et al. [32] reported that the IKDC scores improved from 36.1 ± 12.6 preoperatively to 54.7 ± 20.3 three years after MACI with the Novocart® 3D scaffold in a subgroup of 25 patients with patellar cartilage defects, although better results were observed in a matched subgroup with femoral cartilage defects (mean preoperative score of 33.9 ± 18.1 improving to 71.5 ± 17.4 after 3 years).

Other studies even found comparable results for cohorts with patellofemoral or patellar cartilage defects compared to cohorts with tibiofemoral (TF) defects. Niemeyer et al. [3] reported high clinical success rates in 43 patients with patellar cartilage defects 5 years after third-generation ACI with spheroids (Spherox, Codon, Germany). In their study, the Knee injury and Osteoarthritis Outcome Score (KOOS) scores improved from 54.6 ± 15.7 preoperatively to 82.6 ± 14.0 after 5 years and were just as good as those in 28 patients with femoral defects (60.2 ± 13.9 preoperatively and 81.9 ± 18.6 after five years).

Ebert et al. published two studies on PF MACI that aligned with these favorable results, with the first reporting significantly improved KOOS scores and SF-36 scores as well as pain reduction 2 years after MACI (Genzyme and Maix, Matricel) in 47 patients [26]. A comparison of MACI in the TF joint with MACI in the PF joint two years after implantation also showed similarly good outcomes [24]. Overall, their reported patient-satisfaction levels were high (90.5% and 83.6% in the TF and PF groups, respectively).

Although long-term data on PF MACI are currently lacking, a recent study by Ogura et al. reported good clinical outcomes and a high patient-satisfaction rate after 9 years in a cohort of 58 patients with kissing lesions of the PF joint treated with either first- or second-generation ACI. These findings seem particularly encouraging, given the challenging nature of this patient population.

Data on the specific scaffold as used in this study are sparse in the literature. Niethammer et al. [32] reported good results after 3 years with Novocart® 3D for patellar and femoral cartilage defects. Zak et al. [8] reported mean IKDC scores of 69.8 ± 15.2 in a mixed cohort of 23 patients 2 years after treatment with Novocart® 3D, similar to the current findings after 5–7 years. These results are in concordance with the findings of another study by Niethammer et al. [7], who reported long-term data 10 years after MACI with this scaffold in a mixed cohort of 30 patients.

Overall, the presented clinical outcomes after 5–7 years are comparable to those reported with mixed cohorts and by studies investigating TF MACI. These findings suggest that PF MACI with steps to address maltracking and instability, if necessary, can yield similarly favorable results in the midterm and potentially long term. The other studies reporting MACI data of mixed cohorts indicate that the data obtained the first and second year after MACI often serve as a benchmark for clinical outcomes, and that good results after this time are often are sustained for up to ten years and longer [6, 7, 19–22].

The MRI assessments performed after 5–7 years showed a high mean MOCART score with a high rate of complete defect fills. None of the patients had an effusion, indicating that the transplanted tissue had successfully integrated into the joint. These findings align well with previously published data [8, 9, 18, 24, 26, 33]. Zak et al. [8] published the findings for a mixed but otherwise very similar cohort consisting of 23 patients with a follow-up period of up to two years after MACI using the Novocart® 3D scaffold. They reported that 80.0% of the patients showed complete infills or slight hypertrophy, with another 20.0% showing > 50% infills. The patients’ MOCART score continuously increased from 49.1 ± 16.6 one month after implantation to 71.2 ± 12.9 after one year and 73.2 ± 12.4 after two years. The same group recently repeated these findings in another comparable cohort of 21 patients with mixed PF and TF defects using a different scaffold [33].

Only a few other studies have investigated third-generation MACI in the PF joint with some form of MRI-based assessment. Ebert et al. [26] reported an infill rate of 78.7% after 2 years (46.8% of the patients showed a complete or hypertrophic infill) and a different form of composite MOCART score of 3.2 ± 0.6 out of 4 (ranging from 1 for “poor” to 4 for “excellent”). The same group obtained similar results in MRI assessments in a subgroup of 54 patients with PF MACI that was compared to TF MACI two years after surgery [24]. The reported degree of graft infills rated good or excellent (complete or hypertrophic) was 82%, with an overall MOCART score of 81 in the PF subgroup. Meyerkort et al. [18] also found that 82.0% of the patients showed infills > 50% and a mean MOCART composite score of 3.4 ± 0.1 after 5 years (32.0% with complete infill). In our PF MACI cohort, the MOCART composite scores did not correlate with clinical outcome measures 5–7 years after treatment. This finding aligns with other studies that reported no correlation with the short-, mid-, and long-term MOCART composite scores [8, 9, 33–35].

This study had several limitations. The cohort investigated in this study is quite small, similar to the cohorts in other reports of PF MACI, since a limited number of patients undergo the procedure. There was no control group. Additionally, as is typical in the surgery of the patellofemoral joint, our patient population required a high number of concomitant procedures. This factor could potentially influence the outcomes and should be considered when interpreting the results. The study cohort included both patellar and trochlear defects with a disproportionally a high number of patellar defects versus a low number of trochlear lesions. This imbalance could influence the generalizability of the results, especially regarding the applicability of our findings to trochlear defects. Additionally, no further clinical outcome scoring was performed between the one-year and final follow-up, which is a long time interval. Moreover, the patients started full sports activity 1 year after surgery. Therefore, a decreasing tendency in clinical outcomes before the final follow-up cannot be fully excluded; however, such a tendency is very unlikely considering the findings reported in various other studies. MRI was performed only at the final follow-up, and no baseline MRI was performed after surgery. However, the reported MOCART score at the final follow-up was comparable with the values obtained after 2 years of follow-up reported by Zak et al., underlining the preservation of the reported cartilage tissue quality at midterm follow-up.

## Conclusions

MACI for cartilage defects of the patella demonstrates good midterm clinical results with a significant reduction of pain, improvement in function, and high patient satisfaction. These clinical findings are supported by radiological evidence from MRI observations of cartilage repair tissue.

## Data Availability

The datasets generated and analyzed during the current study are available from the corresponding author on reasonable request.

## References

[1] Minas T (2012) A primer in cartilage repair. J Bone Joint Surg Br 94(11 Suppl A):141–146. 10.1302/0301-620X.94B11.30679 10.1302/0301-620X.94B11.30679 23118403

[2] Colombini A, Libonati F, Lopa S, Peretti GM, Moretti M, de Girolamo L (2023). Autologous chondrocyte implantation provides good long-term clinical results in the treatment of knee osteoarthritis: a systematic review. Knee Surg Sports Traumatol Arthrosc.

[3] Niemeyer P, Laute V, Zinser W (2020). Clinical outcome and success rates of ACI for cartilage defects of the patella: a subgroup analysis from a controlled randomized clinical phase II trial (CODIS study). Arch Orthop Trauma Surg.

[4] Niemeyer P, Albrecht D, Aurich M (2023). Empfehlungen der AG Klinische Geweberegeneration zur Behandlung von Knorpelschaden am Kniegelenk. Z Orthop Unfall.

[5] Vogelmann T, Roessler PP, Buhs M (2023). Long-term cost-effectiveness of matrix-associated chondrocyte implantation in the German health care system: a discrete event simulation. Arch Orthop Trauma Surg.

[6] Ebert JR, Fallon M, Wood DJ, Janes GC (2021). Long-term prospective clinical and magnetic resonance imaging-based evaluation of matrix-induced autologous chondrocyte implantation. Am J Sports Med.

[7] Niethammer TR, Altmann D, Holzgruber M (2020). Patient-reported and magnetic resonance imaging outcomes of third-generation autologous chondrocyte implantation after 10 years. Arthroscopy.

[8] Zak L, Albrecht C, Wondrasch B (2014). Results 2 years after matrix-associated autologous chondrocyte transplantation using the novocart 3d scaffold: an analysis of clinical and radiological data. Am J Sports Med.

[9] Iordache E, Robertson EL, Hirschmann A, Hirschmann MT (2021). Typical MRI-pattern suggests peak maturation of the ACI graft 2 years after third-generation ACI: a systematic review. Knee Surg Sports Traumatol Arthrosc.

[10] Marlovits S, Singer P, Zeller P, Mandl I, Haller J, Trattnig S (2006). Magnetic resonance observation of cartilage repair tissue (MOCART) for the evaluation of autologous chondrocyte transplantation: determination of interobserver variability and correlation to clinical outcome after 2 years. Eur J Radiol.

[11] Trattnig S, Ba-Ssalamah A, Pinker K, Plank C, Vecsei V, Marlovits S (2005). Matrix-based autologous chondrocyte implantation for cartilage repair: noninvasive monitoring by high-resolution magnetic resonance imaging. Magn Reson Imaging.

[12] Domayer SE, Welsch GH, Dorotka R (2008). MRI monitoring of cartilage repair in the knee: a review. Semin Musculoskelet Radiol.

[13] Bellamy N, Buchanan WW, Goldsmith CH, Campbell J, Stitt LW (1988). Validation study of WOMAC: a health status instrument for measuring clinically important patient relevant outcomes to antirheumatic drug therapy in patients with osteoarthritis of the hip or knee. J Rheumatol.

[14] Irrgang JJ, Anderson AF, Boland AL (2001). Development and validation of the international knee documentation committee subjective knee form. Am J Sports Med.

[15] Ware JE, Gandek B (1998). Overview of the SF-36 health survey and the international quality of life assessment (IQOLA) project. J Clin Epidemiol.

[16] Hirschmuller A, Baur H, Braun S, Kreuz PC, Sudkamp NP, Niemeyer P (2011). Rehabilitation after autologous chondrocyte implantation for isolated cartilage defects of the knee. Am J Sports Med.

[17] Filardo G, Kon E, Andriolo L, Di Martino A, Zaffagnini S, Marcacci M (2014). Treatment of "patellofemoral" cartilage lesions with matrix-assisted autologous chondrocyte transplantation: a comparison of patellar and trochlear lesions. Am J Sports Med.

[18] Meyerkort D, Ebert JR, Ackland TR (2014). Matrix-induced autologous chondrocyte implantation (MACI) for chondral defects in the patellofemoral joint. Knee Surg Sports Traumatol Arthrosc.

[19] Aldrian S, Zak L, Wondrasch B (2014). Clinical and radiological long-term outcomes after matrix-induced autologous chondrocyte transplantation: a prospective follow-up at a minimum of 10 years. Am J Sports Med.

[20] Ebert JR, Fallon M, Ackland TR, Janes GC, Wood DJ (2020). Minimum 10-year clinical and radiological outcomes of a randomized controlled trial evaluating 2 different approaches to full weightbearing after matrix-induced autologous chondrocyte implantation. Am J Sports Med.

[21] Gille J, Behrens P, Schulz AP, Oheim R, Kienast B (2016). Matrix-associated autologous chondrocyte implantation: a clinical follow-up at 15 years. Cartilage.

[22] Kreuz PC, Kalkreuth RH, Niemeyer P, Uhl M, Erggelet C (2019). Long-term clinical and MRI results of matrix-assisted autologous chondrocyte implantation for articular cartilage defects of the knee. Cartilage.

[23] Niethammer TR, Altmann D, Holzgruber M, Goller S, Fischer A, Muller PE (2021). Third generation autologous chondrocyte implantation is a good treatment option for athletic persons. Knee Surg Sports Traumatol Arthrosc.

[24] Ebert JR, Schneider A, Fallon M, Wood DJ, Janes GC (2017). A comparison of 2-year outcomes in patients undergoing tibiofemoral or patellofemoral matrix-induced autologous chondrocyte implantation. Am J Sports Med.

[25] Gigante A, Enea D, Greco F (2009). Distal realignment and patellar autologous chondrocyte implantation: mid-term results in a selected population. Knee Surg Sports Traumatol Arthrosc.

[26] Ebert JR, Fallon M, Smith A, Janes GC, Wood DJ (2015). Prospective clinical and radiologic evaluation of patellofemoral matrix-induced autologous chondrocyte implantation. Am J Sports Med.

[27] Kreuz PC, Steinwachs M, Erggelet C (2007). Classification of graft hypertrophy after autologous chondrocyte implantation of full-thickness chondral defects in the knee. Osteoarthritis Cartilage.

[28] Niethammer TR, Loitzsch A, Horng A (2018). Graft hypertrophy after third-generation autologous chondrocyte implantation has no correlation with reduced cartilage quality: matched-pair analysis using t2-weighted mapping. Am J Sports Med.

[29] Pietschmann MF, Niethammer TR, Horng A (2012). The incidence and clinical relevance of graft hypertrophy after matrix-based autologous chondrocyte implantation. Am J Sports Med.

[30] Niemeyer P, Pestka JM, Kreuz PC (2008). Characteristic complications after autologous chondrocyte implantation for cartilage defects of the knee joint. Am J Sports Med.

[31] Niethammer TR, Pietschmann MF, Horng A (2014). Graft hypertrophy of matrix-based autologous chondrocyte implantation: a two-year follow-up study of NOVOCART 3D implantation in the knee. Knee Surg Sports Traumatol Arthrosc.

[32] Niethammer TR, Gallik D, Chevalier Y (2021). Effect of the defect localization and size on the success of third-generation autologous chondrocyte implantation in the knee joint. Int Orthop.

[33] Zak L, Kleiner A, Albrecht C, Tichy B, Aldrian S (2021). Third-generation autologous chondrocyte implantation at the knee joint using the igor scaffold: a case series with 2-year follow-up. Orthop J Sports Med.

[34] de Windt TS, Welsch GH, Brittberg M (2013). Is magnetic resonance imaging reliable in predicting clinical outcome after articular cartilage repair of the knee? A systematic review and meta-analysis. Am J Sports Med.

[35] Ebert JR, Robertson WB, Woodhouse J (2011). Clinical and magnetic resonance imaging-based outcomes to 5 years after matrix-induced autologous chondrocyte implantation to address articular cartilage defects in the knee. Am J Sports Med.

